# Excision of the Inferior Maxilla

**Published:** 1872-12

**Authors:** Lizars Lizars


### ARTICLE IX.

### *Excision of the Inferior Maxilla.*

BY LIZARS LIZARS.

John N——, aged thirty-seven, a native of Glasgow, Scot land, with sandy hair and whiskers, blue eyes, and florid complexion, and well nourished. Had suffered in youth from strumous abscesses of the glandulæ concatinutæ, the cicatrices being still visible. After this he enjoyed excellent health until recently. Never had syphilis, nor was mercurialized as far as he knew, though from his breath, and the state of his teeth and gums, I believe he had. He first consulted me last summer on account of an enlargement of the left side of the inferior maxilla. The tumor was smooth and even externally, and extended from about an inch below the zygoma to a line perpendicular to the angle of the mouth, and was slightly nodulated along the lower margin of the horizontal ramus, as also along its inner surface, where, by its projection inwards, it pushed the tongue slightly to the right side, and interfered with speech. The skin over the parts supplied by the mental branch of the inferior dental was devoid of sensation, and the tumor was painless on manipulation and at all other times.

Unwilling to submit the patient to so severe an operation as excision without an attempt to reduce it by medication, I resorted for a few months to the use of iodides, bromide, and counter-irritants; but the tumor continuing to increase, I was at length forced to operate. Consequently, on the 19th of February, 1872, assisted by Professor Bethtue and two other medical men, the patient being fully under the influence of chloroform, I operated in the manner above described, using the handle (bone) of the scalpel and my fingers as much as possible. The proximal end of the facial and inferior dental were the only vessels requiring ligature, the others being occluded by torsion. The wound having been swabbed out with a solution of carbolic acid, and exposed to the air till all oozing ceased, was accurately adjusted, and kept together by silver wire sutures, pad, and bandage. The patient then rose from the table, got into bed, and immediately asked for food with a pretty clear voice. *At this time and ever since he has had full control of all the muscles supplied by the portio dura.* On the third day after the operation he left his bed. In ten days the wound was healed, with the exception of a small opening at its posterior extremity, through which saliva dribbled away for a few days, but was arrested by the application of nitric acid, after which it rapidly closed. The flow of saliva, I am certain, must have been due either to the existence of the socia parotidis or the division of some of the racinus of the main gland. The only other annoyance I had to contend with was a slight attack of erythema of the left side of the neck, and the formation of a couple of small abscesses at the seat of the old cicatrices above mentioned. The tincture of the muriate of iron externally, the lancet, and poultices, with tonics and generous diet, soon rid me of these, and the patient returned to his office in one month from the date of operation.

In describing this operation I have adhered strictly to what was done. I may now add that in cases where the bone is too dense to be divided by pliers, the chain keys or the metacarpal saw can be easily used to divide it in whole or in part; and that should the tumor prove too large to be removed by the single incision, the surgeon has the option of making fresh incisions from any point of the first, either upwards or downwards, as the exigencies of the case may demand. One from the angle of the mouth downward would, I believe, be the best, as it would divide the smallest number of branches of the facial.

When the patient is a man I can see no objection to this mode of operation. (I have shown N——'s face to medical men and others, but so fully do his whiskers cover the scar, that most have failed to discover the nature of the case.) In the case of women, some may urge that the old line of incision would be the less apparent. In reply, let me ask which is preferable, a simple scar across the face, but full power of all the muscles of expression, or a scar which must show more or less a staring eye, a mouth whose expression is gone, and the saliva probably dribbling from one corner of it? I firmly believe every woman of ordinary sense would prefer the former.

Since operating, I have been able to consult Heath and Guérin, and find that Beaumont and Huguier both operated by a *curved* incision from the angle of the mouth towards the ear, Huguier ending his at the mastoid process. The direction of the curve is not given, neither is the amount of paralysis mentioned, and all that is claimed is that *the eyelids* were unaffected.—*London Lancet.*

